# Si Shen Wan Regulates Phospholipase C**γ**-1 and PI3K/Akt Signal in Colonic Mucosa from Rats with Colitis

**DOI:** 10.1155/2015/392405

**Published:** 2015-07-27

**Authors:** Duan-yong Liu, Rong Xu, Min-fang Huang, Hong-yan Huang, Xin Wang, Yong Zou, Hai-yang Yue, Hai-mei Zhao

**Affiliations:** ^1^Science and Technology College, Jiangxi University of Traditional Chinese Medicine, Nanchang, Jiangxi 330025, China; ^2^Department of Postgraduate, Jiangxi University of Traditional Chinese Medicine, Nanchang, Jiangxi 330004, China; ^3^School of Basic Medical Sciences, Jiangxi University of Traditional Chinese Medicine, Nanchang, Jiangxi 330004, China

## Abstract

The present study explored the feasible pathway of Si Shen Wan (SSW) in inhibiting apoptosis of intestinal epithelial cells (IECs) by observing activation of phospholipase C*γ*-1 (PLC-*γ*1) and PI3K/Akt signal in colonic mucosa from rats with colitis. Experimental colitis was induced by 2,4,6-trinitrobenzene sulfonic acid (TNBS) in the Sprague-Dawley rats. After SSW was administrated for 7 days after TNBS infusion, western blot showed an increment in levels of PI3K, p-Akt, and IL-23 and a decrement in levels of PLC-*γ*1 and HSP70 in colonic mucosal injury induced by TNBS. Meanwhile, assessments by ELISA revealed an increment in concentrations of IL-2, IL-6, and IL-17 and a reduction in level of TGF-*β* after TNBS challenge. Impressively, treatment with SSW for 7 days significantly attenuated the expressions of PI3K and p-Akt and the secretion of IL-2, IL-6, IL-17, and IL-23 and promoted the activation of PLC-*γ*1, HSP70, and TGF-*β*. Our previous studies had demonstrated that SSW restored colonic mucosal ulcers by inhibiting apoptosis of IECs. The present study demonstrated that the effect of SSW on inhibiting apoptosis of IECs was realized probably by activation of PLC-*γ*1 and suppression of PI3K/Akt signal pathway.

## 1. Introduction

Inflammatory bowel disease (IBD) is an immunologically mediated chronic intestinal disorder, including ulcerative colitis and Crohn disease, which is characterized by bouts of severe intestinal inflammation and colonic mucosal ulcer [1, 2]. Although the exact etiology and pathogenesis of IBD still remains unclear, growing studies had indicated that intestinal epithelial cells (IECs) apoptosis played a significantly important role in the occur and procession of IBD [3, 4]. Excessive apoptosis with insufficient proliferation can disrupt intestinal mucosal integrity and barrier function and lead to injury of colonic mucous epithelium, ulcerative formation, and inflammatory cell infiltration and other changes associated with colitis [5, 6]. Therefore, inhibited excessive apoptosis of IECs is probably a necessary method to ameliorated colonic mucosal injury.

As a famous traditional Chinese herbal medicine formula, Si Shen Wan (SSW) is a classic prescription treated chronic colitis. In our previous studies, many evidences had proved that different dosages of SSW restored colonic mucosal ulcers induced by 2,4,6-trinitrobenzene sulfonic acid (TNBS) and dextran sulphate sodium salt (DSS); meanwhile, we found that SSW regulated colonic epithelial cell cycle and inhibited apoptosis of intestinal epithelial cells (IECs) from animals with colitis by intragastric administration. Furthermore, we observed that the antiapoptotic effect of SSW was probably realized by inhibiting mRNA expression of apoptosis-related molecules in p38 MAPK signal pathway. All results had demonstrated that SSW effectively treated colitis by inhibiting excessive apoptosis of IECs [7–9].

Although the molecular basis of IECs' apoptosis is unclear, it is known that cellular death of IECs is controlled by the intrinsic and extrinsic pathway. The extrinsic pathway activated by ligand-induced cell surface receptor (as tumor necrosis factor receptor 1 (TNFR1) and Fas) and the intrinsic mitochondrial apoptosis occurred by apoptotic-related proteins or signal pathway (as mitogen-activated protein kinases (MAPKs), phospholipase C *γ*-1 (PLC-*γ*1) signal, phosphatidylinositol 3-kinase (PI3K)/Akt pathway, etc.) activation [5, 10].

PLC-*γ*, including two forms as PLC-*γ*1 and PLC-*γ*2, is a member of the family of phosphoinositide-specific PLCs (PI-PLC). PLC-*γ*1 is widely distributed in various kinds of tissues (e.g., colonic mucosa, intestine, lung, liver, etc.) [11]. PLC-*γ*1 plays an important protective role in cells apoptosis induced by H_2_O_2_ or ultraviolet C [12, 13]. Remarkably, elevated PLC-*γ*1 expression or activation of PLC-*γ*1 alone is sufficient to suppress cells apoptosis and involved their downstream targets (phosphatidylinositol 3-kinase (PI3K)/Akt signal proteins) of PLC-*γ*1 activation to mediate the protective effects [12, 13]. The present study sought to explore the molecular mechanism of SSW protecting apoptosis of IECs by observing activation of PLC-*γ*1 protein and their downstream signaling factors.

## 2. Materials and Methods

### 2.1. Animals

According to the Guidelines of Jiangxi University of Traditional Chinese Medicine (TCM), Animal Research Committee, forty Male Sprague-Dawley rats (weighing 200 to 220 g, certificate number: SCXK 2012-0001) were purchased from the Animal Center of Peking University Health Science Center. All rats were housed in a special room with a humidity of 50% ± 5% and lived in a 12 h light/dark cycle at 20 ± 2°C. Throughout the whole experiment, all animals were freely provided standard diet and water ad libitum. The rats were acclimatized for 3 days before experiments. The experimental protocols were supported by Jiangxi University of TCM Biomedical Ethics Committee, Experimental Animal Ethics Branch (JZ2014-79).

### 2.2. Drugs

Si Shen Wan (SSW) (batch number: 12080004) was purchased from Tongrentang (TRT) Pharma (Beijing, China). Mesalazine (batch number: 130407) was provided by Sunflower Pharma (Jiamusi, China). 2,4,6-Trinitrobenzene sulfonic acid (TNBS) (batch number: p2297) was gotten from Sigma (St. Louis, MO, USA).

### 2.3. Experimental Colitis Induced by TNBS

The animal model was induced according to the reported article with a little change [14, 15]. Briefly, the experimental colitis was induced by TNBS in rats. After 12 h of absolute diets, rat was lightly anesthetized with pentobarbital (60 mg/kg, i.p.) and received clyster with a dose of 100 mg/kg TNBS solution. The fresh solution was prepared in the light of the prescription (100 mg·kg^−1^ body TNBS was dissolved in 0.30 mL of 50% ethanol), and injected into the colon 8 cm proximal to the anus by a plastic hose tube whose diameter is 2 mm. And then, the rat was kept a head-down position for 15 min to full with the whole colon.

### 2.4. Experimental Protocol

According to calculating by body surface area between human and rat, we designed dosage of SSW and projected the experimental protocol on the basis of our previous studies [7–9]. Ten animals were in each group. The total 40 rats were randomly assigned into four groups: the Normal group (Normal; rats were induced and administrated by physiological saline), the TNBS 8d group (TNBS 8d; rats were induced by TNBS and 24 h thereafter physiological saline was administrated by gavage for 7 days), the TNBS 8d + SSW group (TNBS 8d + SSW; rats were induced by TNBS and 24 h thereafter SSW was administrated at 2.5 g/kg everyday by gavage for 7 days), and the TNBS 8d + Mesalazine group (TNBS 8d + Mesalazine; rats were induced by TNBS and 24 h thereafter Mesalazine was administrated at 300 mg/kg everyday by gavage for 7 days). At the end of treatment on day 9, all rats were fasted for 12 h and then sacrificed by cervical dislocation after anesthesia with intraperitoneally administrated urethane (2.0 g/kg). The whole colon was separated rapidly and opened longitudinally along colonic mesentery to clear its contents on an ice block. The colon was divided into two parts. One part was used to prepare colonic tissue homogenate, and the other was preserved in −80°C to Western blot analysis.

### 2.5. Enzyme-Linked Immunosorbent Assay

Colonic tissues were lysed in RIPA buffer (50 mM Tris-HCl at pH 7.4, 150 mM sodium chloride, 1% NP-40, 0.5% sodium deoxycholate, and 0.1% sodium dodecyl sulfate) with protease and phosphate inhibitor cocktail (Merk, Ashland, MA, USA) using a sonicator. Crude lysates were centrifuged at 19357 g for 20 min. The supernatant (*n* = 10) was used to measure the level of interleukin- (IL-) -2, IL-6, IL-17, and transforming growth factor- (TGF-) *β*1 by following the manufacturer instructions. Thecytokine ELISA kits (IL-2, IL-6, IL-17, and TGF-*β*1) were purchased from eBioscience (San Diego, CA).

### 2.6. Western Blot Analysis

Western blot analysis (*n* = 6 for each group) was performed as described previously [16]. Briefly, the protein concentration of the supernatants was measured by bicinchoninic acid (BCA) assay. The protein (10–30 *μ*g) was separated by 9–12% sodium dodecyl sulphate- (SDS-) polyacrylamide gel electrophoresis for 1.5 h at 80 V and then blotted on polyvinylidene fluoride (PVDF) membranes (Amersham Pharmacia, Little Chalfont, UK). Membranes were treated with 5% nonfat dry milk in phosphate-buffered saline containing 0.01% Tween 20 (TBST) to block any nonspecific antibody binding sites and then probed overnight at 4°C with anti-PLC-*γ*1, phospho-PLC-*γ*1, -Akt, anti-phospho-Akt, PI3K, IL-23, HSP70, and GAPDH (Abcam, Cambridge, UK) monoclonal antibody at 1 : 1000–1 : 4,000 dilution with TBST containing 5% nonfat dry milk. After washing with TBST, the membranes were incubated for 1 h at room temperature with secondary HRP conjugated antibody (Dako, Glostrup, Denmark, 1 : 4,000) and visualized using ECL detection kit (Amersham Pharmacia, Uppsala, Sweden). Finally, bands were quantified using Image-Pro Plus 5.0 software (Media Cybernetic, Bethesda, MD, USA).

### 2.7. Statistical Analysis

All statistical analyses were performed with SPSS version 15.0 (SPSS Inc., Chicago, IL, USA) for Windows. Measurement data were expressed as mean ± SD and analyzed using an analysis of variance (ANOVA) followed by the Tukey test for comparison of >2 condition. In this study, a difference was considered significant when *P* < 0.05.

## 3. Results

### 3.1. SSW Decreased the Levels of IL-2, IL-6, IL-17, and IL-23, and Increased the Level of TGF-*β* in Colonic Mucosa

**Figure 1:**
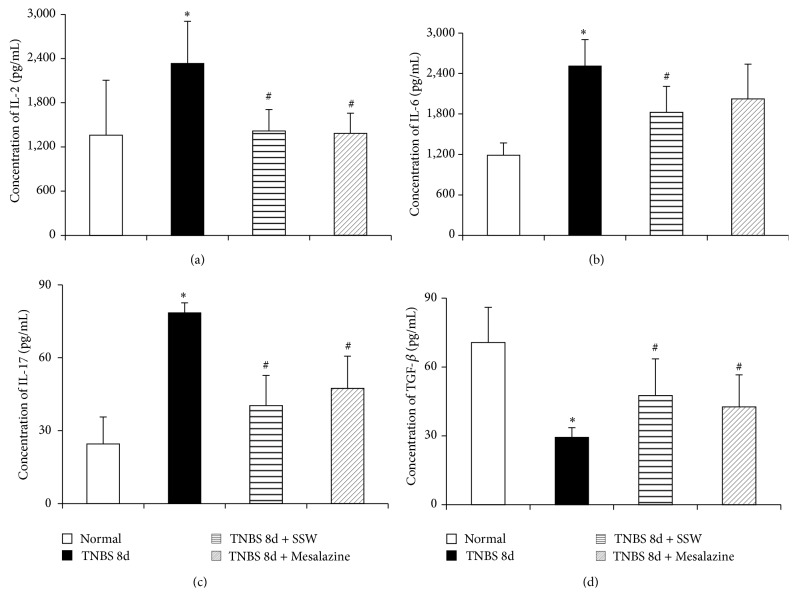
Concentration of IL-2, IL-6, IL-17, and TGF-*β* in colonic mucosa. (a) Concentration of IL-2 in colonic mucosa from different groups. (b) Concentration of IL-6 in colonic mucosa from different groups. (c) Concentration of IL-17 in colonic mucosa from different groups. (d) Concentration of TGF-*β* in colonic mucosa from different groups. Data were mean ± SD (*n* = 10). ^*^ *P* < 0.05 versus Normal group; ^#^ *P* < 0.05 versus TNBS 8d group.

After 7 days that experimental colitis was induced by TNBS and proinflammatory cytokines were released in inflammatory zone. In Figures 1(a), 1(b), 1(c), 3(a) and 3(b), the levels of IL-2, IL-6, IL-17, and IL-23 in colonic mucosa from colitis rats in the model group increased remarkably when they were compared with that in the Normal groups (*P* < 0.05). However, the expressions of the four cytokines decreased markedly in the TNBS 8d + SSW group (*P* < 0.05). While in Figure 1(d), compared with in the normal colonic mucosa, TGF-*β* was low expression in colonic mucosa from colitis rats in the model group (*P* < 0.05). But the level of TGF-*β* was higher in the TNBS 8d + SSW groups than that in the TNBS 8d group (*P* < 0.05). Except for IL-6, statistical results of the other three cytokines and TGF-*β* in the Mesalazine group were consistent with the TNBS 8d + SSW group. It is no statistical difference between the TNBS 8d + SSW group and the Mesalazine group.

### 3.2. SSW Elevated Expressions of PLC-*γ*1 and Hsp70 in Colonic Mucosa

**Figure 2:**
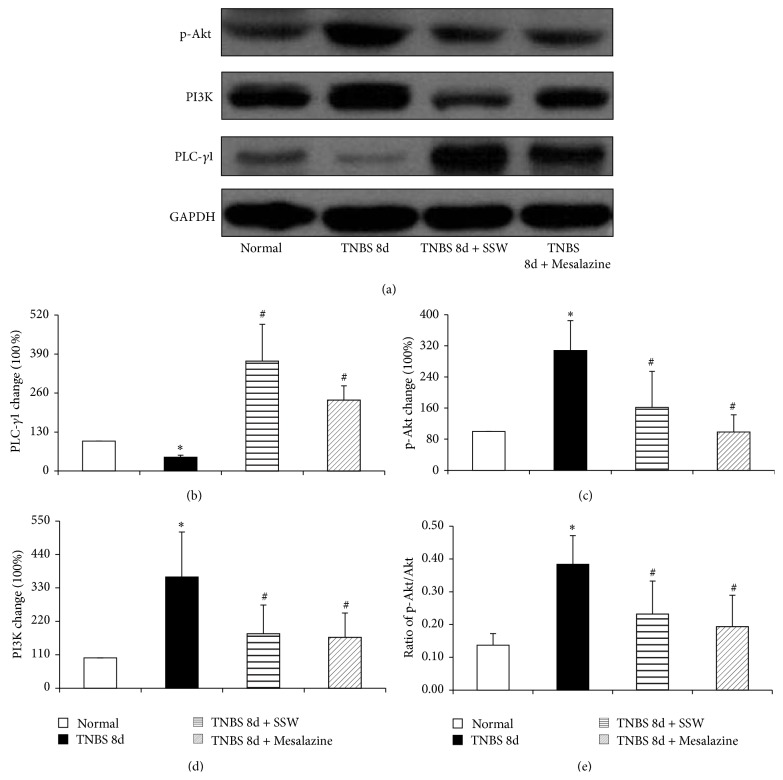
Western blot analysis of PLC-*γ*1, PI3K, and p-Akt. (a) Representative Western blot of PLC-*γ*1, PI3K, p-Akt, and GAPDH (*n* = 6). (b) Quantitative analysis of PLC-*γ*1 protein (*n* = 6). (c) Quantitative analysis of p-Akt protein (*n* = 6). (d) Quantitative analysis of PI3K protein (*n* = 6). (e) Ratio of p-Akt/Akt. Data were mean ± SEM (*n* = 6). ^*^ *P* < 0.05 versus Normal group; ^#^ *P* < 0.05 versus TNBS 8d group.

**Figure 3:**
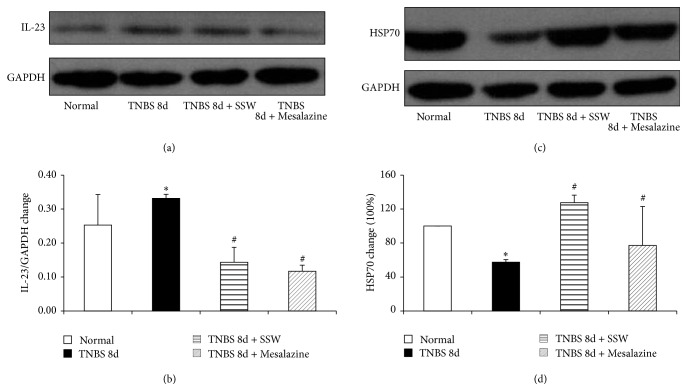
Western blot analysis of IL-23 and HSP70. (a) Representative Western blot of IL-23 and GAPDH (*n* = 6). (b) Quantitative analysis of IL-23 protein (*n* = 6). (c) Representative Western blot of HSP70 and GAPDH (*n* = 6). (d) Quantitative analysis of HSP70 protein (*n* = 6). Data were mean ± SEM (*n* = 6). ^*^ *P* < 0.05 versus Normal group; ^#^ *P* < 0.05 versus TNBS 8d group.

The expressions of PLC-*γ*1 and Hsp70 were assessed by Western blot. In Figures 2(a), 2(b), 3(c), and 3(d), when they were compared with colitis rats without treatment, the expressions of PLC-*γ*1 and Hsp70 were elevated significantly after 7 days of treatment by SSW and Mesalazine or in the Normal group (*P* < 0.05).

### 3.3. SSW Inhibited Expression of PI3K, p-Akt in Colonic Mucosa, and Decreased the Ratio of p-Akt/Akt

The PI3K is one of downstream proteins of PLC-*γ* signal [17]. The expression of PI3K and the activation of phosphor-Akt (p-Akt) were analyzed by Western blot. As seen in Figures 2(a), 2(c) and 2(d), we found that the expressions of PI3K and p-Akt were distinctly heightened in the colonic mucosa from colitis rats without treatment when they compared with that in normal rats (*P* < 0.05). Nevertheless, it was very worthy that their expressions were reduced obviously in the colitis rats treated by 7 days of SSW and Mesalazine (*P* < 0.05). In the meantime, we computed the ratio of p-Akt/Akt to show the extent of phosphor-Akt in the present study (*P* < 0.05). The ratio was lower after 7 days of treatment with SSW and Mesalazine than in the TNBS 8d groups (*P* < 0.05) (Figure 2(e)). The results declared that the phosphorylation of Akt was inhibited after treatment by SSW.

## 4. Discussion

Increased apoptosis of colonic epithelial cells is an important character in the pathological change of IBD [3, 4]. In our previous studies, we had proved that SSW effectively treated TNBS- or DSS-induced experimental colitis by inhibiting IECs apoptosis [7–9]. In the present study, SSW significantly elevated expression of PLC-*γ* protein in the colonic mucosa from colitis rats and, meanwhile, inhibited activation of PI3K and p-Akt. Both of the two results hinted that the effect of SSW inhibiting IECs apoptosis was probably related to the activation of PLC-*γ* signal and PI3K/Akt pathway.

As an important intermediary of growth factor signal pathway in the family of phosphoinositide-specific PLCs (PI-PLC), PLC-*γ*1 is double identity of important molecule in which PLC-*γ*1 can regulate and control cell proliferation and apoptosis [13, 18–20]. Growing evidences have demonstrated that PLC-*γ*1 plays a negative control role in the process of cell apoptosis. Some researchers had observed that obvious apoptotic morphology and the percentage of apoptotic cells were dramatically exhibited and rose when the PLC-*γ*1 signal pathway was inhibited by blocker [21]. In present and previous studies, we had found that low expression of PLC-*γ*1 and increased IECs apoptosis were simultaneously seen in the same animal model induced by TNBS, while SSW effectively decreased IECs apoptosis and improved expression of PLC-*γ*1 to alleviate colonic mucosal injury of rats with experimental colitis. The results had shown that the protective effect of SSW on damaged colonic mucosa was realized by promoting PLC-*γ*1 activation to inhibit IECs apoptosis [7–9]. The probable mechanism of PLC-*γ*1 signal is that proteolytic cleavage of PLC-*γ*1 is blocked by overexpression of Bcl-2, which can prevent apoptosis at a step during the activation of caspase family proteases such as caspase-9 and caspase-3 [22]. This point was in accord with our previous study showing that SSW heightened expression of Bcl-2 mRNA in colonic mucosa from rats with colitis [7].

**Figure 4:**
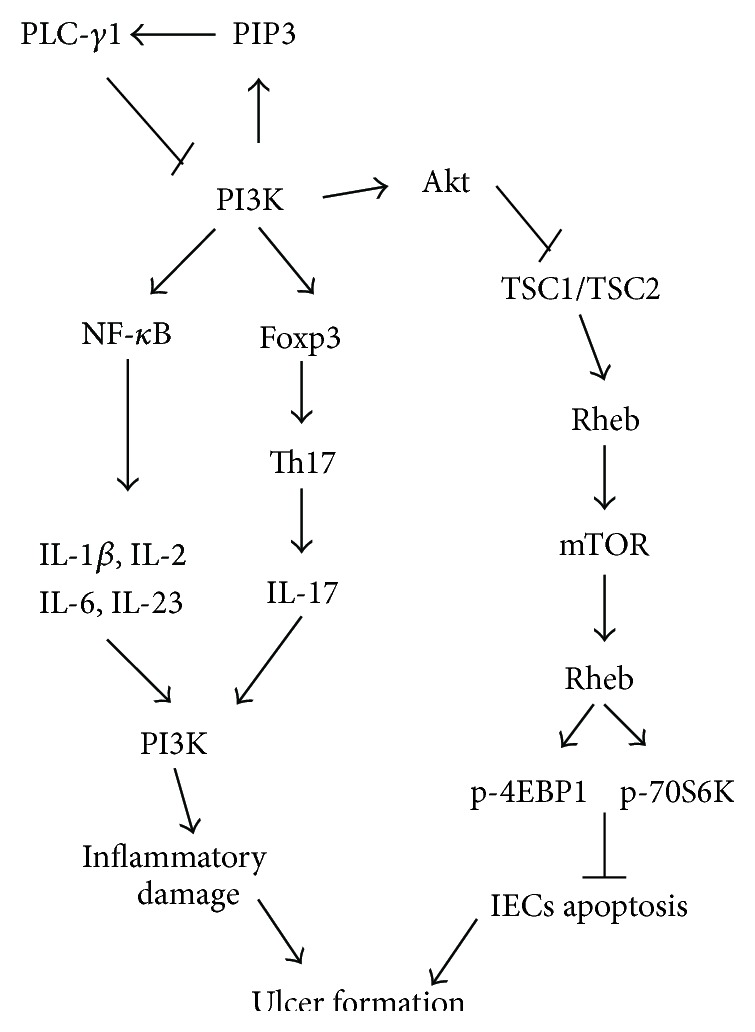
Schematic illustration of PLC-*γ*1 and PI3K/Akt signal.

As shown in Figure 4, many studies had indicated that PLC-*γ*1 pathway and PI3K-Akt pathway interacted with each other. However it is not known whether PLC-*γ*1 directly binds to Akt and well known that PI3K may affect the translocation of PLC-*γ*1 by generating PI3P [23] and that both PLC-*γ*1 and phosphatidylinositol 3-kinase (PI3K)/Akt signal are essential mediators of cellular processes such as growth, apoptosis, and cell proliferation [24, 25].

Recently, it is a hotspot that PI3K/Akt signal pathway, including PI3K, Akt, phosphatase, and tens in homolog on chromosome10 (PTEN), tuberous sclerosis complex 1/2 (TSC1/2) and target of rapamycin (mTOR), plays central roles in regulating cell growth and apoptosis. PIP3 is generated by activation of PI3K and can interact with PI3K to activate Akt by phosphorylation on Ser473 in a hydrophobic motif and Thr308 in the activation loop of the kinase domain. As a candidate, mTOR is activated by inhibiting TSC 1/2 or phosphorylated by phosphoinositide dependent protein kinase 1 (PDK1) [26, 27]. As a core controller of cyclin synthetic, activated mTOR may phosphorylate 4EBP1 and activate S6-kinase (p70S6 K) to enhance protein translation following Hsp70, Protor, and Deptor and finally inhibit cell apoptosis with independent of p53 [26, 28, 29]. Previous studies had shown that mesalazine effectively treated mice with Dss-induced colitis and affected the PI3K/Akt signal [30, 31].

Meanwhile, the primary function of PI3K-driven NF-*κ*B activation is to promote expression and secretion of cytokines (IL-6, IL-23, IL-2, etc.) and chemokines [32]. Luyendyk and his colleagues had found that inhibition of PI3K/Akt pathway decreased transcriptional level of downstream cytokines (IL-6, IL-1*β*, and IL-2) to alleviate severity of inflammation [33]. And that PI3K signal can inhibit expression of Foxp3 and restrain activation of regulatory of T cell and activate Th17 cell. Activatory Th17 cells secreted abundant IL-17 and IL-23 contributed to the occurrence of several autoimmune diseases such as IBD and rheumatoid arthritis [34, 35].

In the present study, PI3K and p-Akt proteins were activated in the colonic mucosa from colitis rats without treatment; in the meantime, the levels of IL-2, IL-6, IL-17, and IL-23 were increased, and the expressions of HSP70 and TGF-*β* were decreased. This hinted that in the course of TNBS-induced colitis, activated PI3K/Akt signal pathway possibly heightened transcription of NF-*κ*B, and then promoted to secrete proinflammatory cytokines (including IL-2, IL-6, IL-17, and IL-23), inhibited expressions of HSP70 and TGF-*β*, and finally led to inflammatory injury of colonic mucosa. Interestingly, PI3K signal was inactivated after treatment with SSW, the ratio of p-Akt/Akt was reduced, the secretions of those cytokines were downregulated, and the levels of HSP70 and TGF-*β* were elevated. These evidences indicated that SSW had inactivated PI3K/Akt signal pathway. Meanwhile, we found that there was no difference between SSW and Mesalazine to regulate expression of these cytokines and proteins. The results hinted that SSW had some extent similar to Mesalazine on curative effect and probable pharmacological mechanism. To sum up, SSW can restrain PI3K/Akt signal and activated PLC-*γ*1 protein in colonic mucosa from colitis rats. As a complex system, abundant active constituents of SSW and their pharmacological action are uncertain. However, several known constituents (including evodiamine and rutaecarpine) treated experimental colitis and controlled apoptosis by regulating PI3K/Akt signal (as evodiamine) [36, 37]. Nevertheless, a definite element of SSW to treat IBD by PLC-*γ*1 and PI3K/Akt signal has not been reported. Consequently, the next step would be very significant to seek out effective component of SSW.

## 5. Conclusions

According to our previous studies, in conclusion, effect of SSW on inhibiting IECs apoptosis is potentially related with activation of PLC-*γ*1 and retardant of PI3K/Akt signal pathway in the therapeutic process of IBD.
